# Inhibition of miR-154 Protects Against Cardiac Dysfunction and Fibrosis in a Mouse Model of Pressure Overload

**DOI:** 10.1038/srep22442

**Published:** 2016-03-01

**Authors:** Bianca C. Bernardo, Sally S. Nguyen, Xiao-Ming Gao, Yow Keat Tham, Jenny Y. Y. Ooi, Natalie L. Patterson, Helen Kiriazis, Yidan Su, Colleen J. Thomas, Ruby C. Y. Lin, Xiao-Jun Du, Julie R. McMullen

**Affiliations:** 1 Baker IDI Heart and Diabetes Institute, Melbourne, 3004, Australia; 2 Department of Physiology, Anatomy and Microbiology, La Trobe University, Bundoora, 3086, Australia; 3 Monash University, Clayton, 3800, Australia; 4 Asbestos Diseases Research Institute, Concorde Hospital, 2139, Australia; 5 Ramaciotti Centre for Genomics and School of Biotechnology and Biomolecular Sciences, University of New South Wales, Sydney, 2052, Australia

## Abstract

Expression of miR-154 is upregulated in the diseased heart and was previously shown to be upregulated in the lungs of patients with pulmonary fibrosis. However, the role of miR-154 in a model of sustained pressure overload-induced cardiac hypertrophy and fibrosis had not been assessed. To examine the role of miR-154 in the diseased heart, adult male mice were subjected to transverse aortic constriction for four weeks, and echocardiography was performed to confirm left ventricular hypertrophy and cardiac dysfunction. Mice were then subcutaneously administered a locked nucleic acid antimiR-154 or control over three consecutive days (25 mg/kg/day) and cardiac function was assessed 8 weeks later. Here, we demonstrate that therapeutic inhibition of miR-154 in mice with pathological hypertrophy was able to protect against cardiac dysfunction and attenuate adverse cardiac remodelling. The improved cardiac phenotype was associated with attenuation of heart and cardiomyocyte size, less cardiac fibrosis, lower expression of atrial and B-type natriuretic peptide genes, attenuation of profibrotic markers, and increased expression of p15 (a miR-154 target and cell cycle inhibitor). In summary, this study suggests that miR-154 may represent a novel target for the treatment of cardiac pathologies associated with cardiac fibrosis, hypertrophy and dysfunction.

Cardiac hypertrophy in response to sustained increases in blood pressure or pressure overload due to aortic stenosis typically leads to adverse cardiac remodelling including left ventricular (LV) dilatation, cardiac fibrosis and the progression to heart failure (HF). HF represents a major global health problem which is becoming worse as the population ages, and the prognosis is worse than that of most cancers^1^. Despite significant advances in cardiovascular medicine, management and surgery, mortality rates remain high, with almost 50% of people diagnosed with HF dying within 5 years^2^,^3^. Thus, there are increased efforts to develop new therapies for the prevention and treatment of HF. We have previously demonstrated that phosphoinositide 3-kinase [PI3K(p110α)] represents a promising target for preserving cardiac function and myocardial viability in a range of heart disease mouse models^4^,^5^,^6^,^7^,^8^. Thus, targeting genes such as microRNAs (miRNAs), that are regulated by the cardioprotective kinase PI3K(p110α), may represent a promising therapeutic approach to improve function of the failing heart.

miRNAs are evolutionary conserved, short strands of RNA that are not transcribed into protein (like typical RNA molecules), but instead, regulate the expression of numerous genes by interacting with specific sites in 3′ untranslated regions of messenger transcripts to prevent protein translation and gene expression^9^,^10^. miRNAs have a crucial role in health and disease^11^, including the development of cardiac hypertrophy and HF^12^,^13^,^14^. The actions of disease-causing miRNAs can be blocked by a class of synthetic locked nucleic acid (LNA)-oligonucleotides, some of which have been shown to improve cardiac function and pathology in preclinical rodent models^15^,^16^,^17^,^18^. The translational potential and safety of miRNA-based therapeutics to patients has been shown in clinical trials for the treatment of hepatitis C virus^19^. Thus, there is promise for the successful development of miRNA-based therapies for the treatment of HF.

In a previous genome-wide transcriptome study we screened for miRNAs in the heart which were regulated by PI3K(p110α), elevated in a diseased setting, and decreased in a protected setting^4^. We subsequently inhibited miRNA candidates (miR-34, miR-34a and miR-652) with LNA-antimiR-based drugs and demonstrated that this approach was associated with reduced pathology and improved cardiac function in mouse models of cardiac disease^15^,^16^,^17^,^18^. The goal of the current study was to target miR-154, a PI3K-regulated miRNA, which had been reported to have a profibrotic role in the human lung^20^. The role of miR-154 in the heart, and more specifically cardiac fibrosis, had not previously been examined.

Cardiac fibrosis leads to stiffness of the heart, and negatively impacts cardiac function leading to HF^21^. With limited effective therapies, treating fibrotic disorders represents a major unmet need^22^,^23^. In the current study, we assessed the therapeutic potential of inhibiting miR-154 in a mouse model with pre-existing pressure overload-induced pathological hypertrophy and cardiac dysfunction due to transverse aortic constriction (TAC). This model is associated with significant cardiomyocyte hypertrophy, cardiac fibrosis and elevated expression of HF molecular markers including atrial and B-type natriuretic peptides (ANP and BNP)^8^,^15^,^16^. We report here, that treatment with LNA-antimiR-154 in a setting of pressure overload was able to i) maintain cardiac function, ii) attenuate hypertrophy (increases in heart and cardiomyocyte size), iii) improve expression of cardiac pathology markers, and iv) prevent a significant increase in cardiac fibrosis.

## Results

### miR-154 expression was increased in the diseased heart and depressed in the protected heart

**Figure 1:**
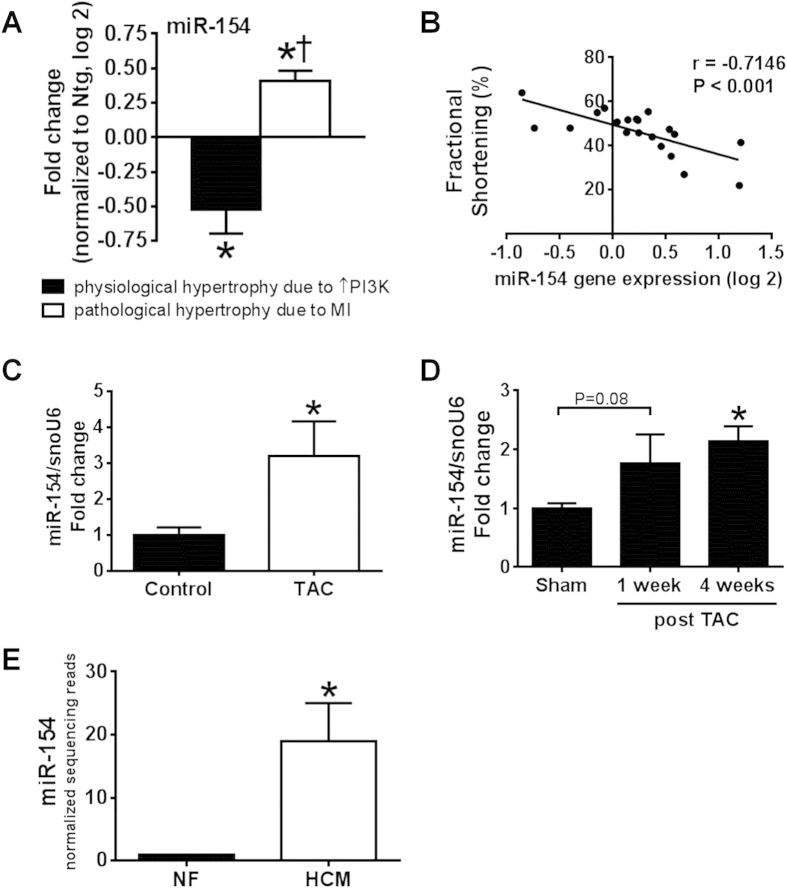
Differential regulation of miRNA-154 in settings of cardiac protection and cardiac stress. (**A**) Gene expression of miR-154 by microarray in mouse hearts from a physiological and pathological setting, normalized to Ntg sham. Data are expressed as mean ± SEM. N = 4/group. *P < 0.05 vs. Ntg, ^†^P < 0.05 vs. physiological setting. (**B**) Linear correlation between miR-154 gene expression (log 2) in hearts from PI3K transgenic mice subjected to sham or MI from microarray analysis and fractional shortening 4. N = 20. P < 0.01 (Spearman’s correlation). (**C**) Gene expression of miR-154 by qPCR in hearts from mice subjected to TAC for 12 weeks compared to un-operated control hearts. Hearts obtained from a previous study^15^. N = 3–5/group. *P < 0.05 (unpaired t-test). (**D**) Gene expression of miR-154 by qPCR in hearts from mice subjected to TAC for 1 and 4 weeks compared to sham hearts. N = 4–6/group. *P < 0.05 vs. Sham. (**E**) Quantification of miRNA-154 in Non-Failing (NF) and Hypertrophic Cardiomyopathy (HCM) human hearts. N = 4–5/group. *P < 0.05 vs. NF. Data taken from supplementary material of full normalized sequencing reads^42^.

Further analysis from our previous microarray screen^4^ identified miR-154 expression to be i) upregulated in ventricular tissues from a mouse model of myocardial infarction (MI) and pathological hypertrophy (Fig. 1A); ii) downregulated in a mouse model of cardioprotection (due to cardiac specific over-expression of PI3K[p110α], Fig. 1A); and iii) inversely correlated with cardiac function (Fig. 1B)^4^. In addition, analysis of miR-154 expression by qPCR in hearts of mice subjected to pressure overload (induced by TAC) for 12 weeks from a previous study^15^ demonstrated that miR-154 is increased in a setting of pathological hypertrophy (Fig. 1C). Furthermore, miR-154 expression in hearts of mice tended to increase following 1 week of pressure overload and was significantly elevated at 4 weeks of pressure overload (Fig. 1D). Finally, by mining and analyzing publically available profiling datasets we found increased expression of miR-154 in hearts from HF patients with hypertrophic cardiomyopathy (compared to a non-failing cohort) (Fig. 1E).

### LNA chemistry had no effect on organ weights in adult mice

First, we compared LNA-control treated mice to saline treated mice for any effects of the LNA chemistry. In adult male sham and unoperated, aged matched C57BL/6 mice, the LNA-control compound had no effect on body weight, tibial length, and organ weights (including the heart, atria, lung, kidney and liver) compared with the same dosing regimen of saline (Supplementary Fig. S1). Thus, sham and unoperated mice administered saline or LNA-control have been combined.

### Inhibition of miR-154 attenuated pathological cardiac remodelling and lung congestion

**Figure 2:**
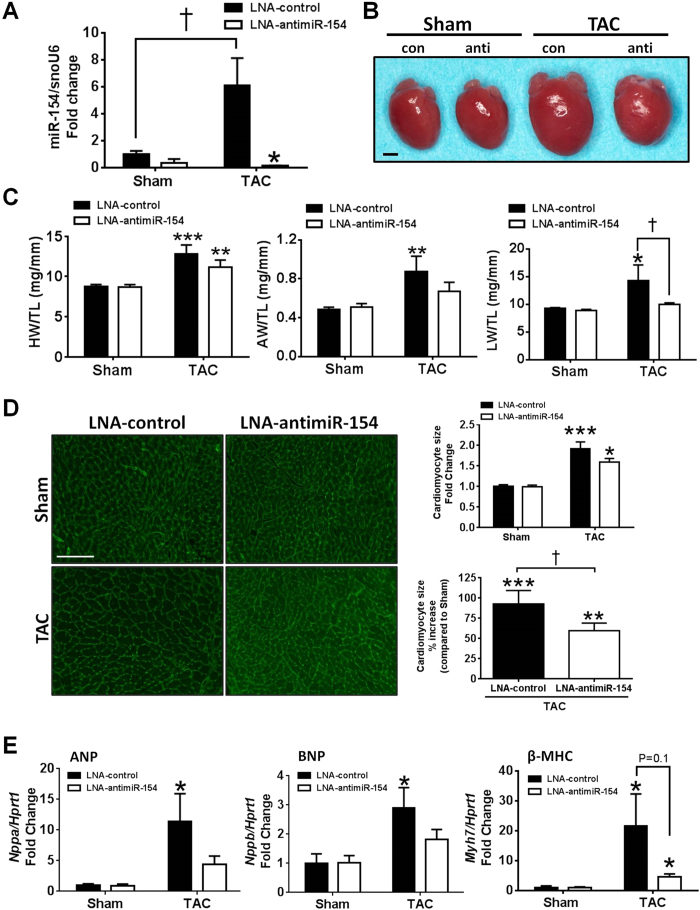
Inhibition of miR-154 protected against pressure overload-induced pathological hypertrophy (**A**) Quantification of miR-154 relative to snoU6 by qPCR. Data are expressed as mean ± SEM. N = 3–6/group. *P < 0.05 vs. LNA-control of the same operation; ^†^P < 0.05. (**B**) Representative hearts from sham LNA-control (con), sham LNA-antimiR-154 (anti), TAC LNA-control (con) and TAC LNA-antimiR-154 (anti) treated mice at dissection. Scale bar = 0.2 cm. (**C**) Graphs of heart weight/tibial length (HW/TL), atrial weight/TL (AW/TL) and lung weight/TL (LW/TL). Data are expressed as mean ± SEM. N = 7–12/group. *P < 0.05 vs. sham of same treatment group; **P < 0.01 vs. sham of same treatment group; ***P < 0.001 vs. sham of same treatment group; ^†^P < 0.05. (**D**) Representative left ventricle cross-sections stained with wheat germ agglutinin (WGA) from sham and TAC LNA-control and LNA-antimiR-154 treated mice and quantification of cell area. Scale bar = 100 μM. Data are expressed as mean ± SEM. Upper graph: N = 3 (sham groups) 7–8 (TAC groups). *P < 0.05 vs. sham of the same treatment group; ***P < 0.001 vs. sham LNA-control. Lower graph: Cardiomyocyte size expressed as a percent increase compared to combined sham groups (as sham LNA-control and sham LNA-antimiR-154 were not different). ***P < 0.001 vs. sham; **P < 0.01 vs. sham; ^†^P < 0.05. (**E**) qPCR analysis of *ANP (Nppa), BNP(Nppb)* and *β-MHC (Myh7)* standardized to *Hprt1* in sham and TAC LNA-control and LNA-antimiR-154 treated mice. Data are expressed as mean ± SEM. N = 3–8 per group. For ANP and BNP: *P≤0.05 vs. sham of the same treatment group (One way ANOVA followed by *Fisher’s* post-hoc test). For β-MHC: *P < 0.05 vs. sham of same treatment group (Mann Whitney nonparametric test for LNA-control sham vs. TAC. Unpaired t-test for LNA-antimiR-154 sham vs. TAC).

Adult mice were subjected to a sham or TAC operation. TAC induced a chronic pressure load on the heart and is associated with progressive pathological hypertrophy and cardiac dysfunction. Following 4 weeks of TAC, left ventricular (LV) remodelling and cardiac dysfunction was confirmed by echocardiography and mice were then subcutaneously administered LNA-control or LNA-antimiR-154 (Supplementary Fig. S2). miR-154 expression increased in TAC LNA-control hearts compared to sham LNA-control hearts, and was inhibited in the hearts of sham and TAC LNA-antimiR-154 treated mice (Fig. 2A). This supports our previous observation of increased expression of miR-154 in the heart following a pathological insult (Fig. 1A,C).

Pathological cardiac hypertrophy together with depressed cardiac function is typically associated with an increase in heart and atrial size, and lung congestion. TAC LNA-control mice displayed an increase in heart weight to tibial length ratio of approximately 47% versus sham mice (Fig. 2B,C; Supplementary Table S1) compared to only a 29% increase in TAC LNA-antimiR-154 treated mice (Fig. 2B,C; Supplementary Table S1). TAC LNA-control mice also displayed an increase in atrial weight to tibial length ratio (AW/TL, ~80% increase, Fig. 2C) and significant lung congestion (lung weight to tibial length ratio [LW/TL] increased ~55%, Fig. 2C, Supplementary Table S1) compared to sham control mice. In contrast, these parameters were not elevated in TAC LNA-antimiR-154 treated mice (Fig. 2C, Supplementary Table S1). Differences in heart size were accompanied by similar differences in cardiomyocyte size (Fig. 2D). Compared with sham mice, myocyte size increased by approximately 93% in TAC LNA-control hearts but only 60% in TAC LNA-antimiR-154 treated hearts (Fig. 2D). TAC-induced hypertrophy is typically associated with an increase in ANP, BNP, and β-myosin heavy chain (β-MHC). ANP and BNP expression was significantly increased in TAC LNA-control hearts but not in TAC LNA antimiR-154 treated hearts (Fig. 2E). β-MHC expression was also elevated in TAC LNA-control hearts and tended to be lower in TAC LNA-antimiR-154 treated hearts (Fig. 2E).

### LNA-antimiR-154 treatment was associated with preserved cardiac function

**Figure 3:**
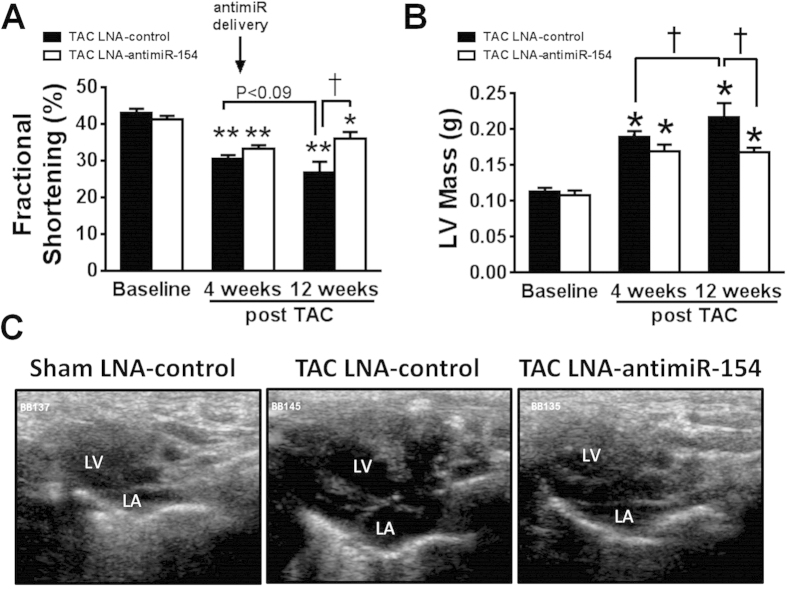
Treatment with LNA-antimiR-154 protected against cardiac dysfunction, LV mass and left atrial enlargement. (**A**) Quantification of fractional shortening at baseline (presurgery), 4 weeks post-TAC (prior to LNA-oligonucleotide delivery), and 8 weeks post-treatment (12 weeks post-TAC). AntimiR delivery commenced at 4 weeks post-TAC (indicated by arrow). Data are expressed as mean ± SEM. N = 7–8/group. Two way repeated measures ANOVA with *Fisher’s* post-hoc test. **P < 0.001 vs. corresponding baseline group; *P < 0.05 vs. corresponding baseline group; ^†^P < 0.001. (**B**) Quantification of left ventricular mass at baseline, 4 weeks post-TAC and 12 weeks post-TAC. Data are expressed as mean ± SEM. N = 7–8/group. Two way repeated measures ANOVA with *Fisher’s* post-hoc test. *P < 0.05 vs. corresponding baseline group; ^†^P < 0.05. (**C**) Representative image of enlarged left atrium (LA; echocardiography: long-axis 2-dimensional image), 8 weeks post-treatment in TAC LNA-control mice and attenuation in TAC LNA-antimiR-154-treated mice.

Following 4 weeks of pressure overload, TAC mice displayed increased LV wall thickness, increased LV mass and depressed cardiac function (fractional shortening, FS, reduced by 20–25% compared with pre-surgery values and sham mice, Fig. 3A,B, Supplementary Table S2). Following 8 weeks of LNA-oligonucleotide treatment, cardiac function in TAC-control mice remained impaired (Fig. 3A, Supplementary Table S2), LV mass further increased (Fig. 3B, Supplementary Table S2) and the left atrium was enlarged as visualized by echocardiography (Fig. 3C). Treatment with LNA-antimiR-154 was associated with more favorable cardiac function post-treatment in TAC LNA-antimiR-154 mice (FS 33% higher compared to TAC LNA-control mice, Fig. 3A, Supplementary Table S2). This was accompanied by improvements in LV dimensions, attenuation of left atrial size and no further increase in LV mass (Fig. 3B,C, Supplementary Table S2).

### Fibrotic and autophagic pathways were enriched for predicted miR-154 targets

**Figure 4:**
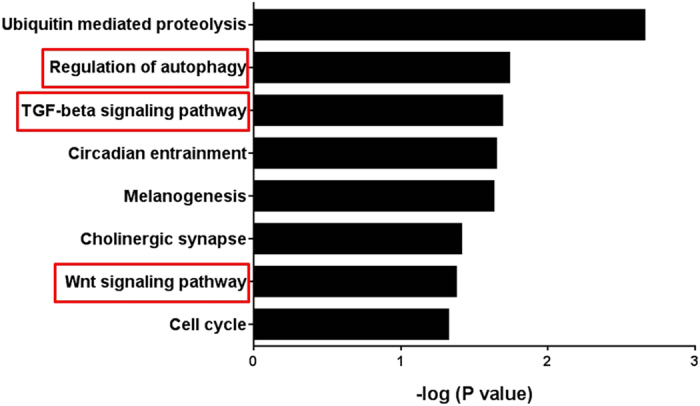
Enriched pathways in miR-154 target genes. TargetScan 6.2 was used to identify the pathways that were enriched in miR-154 predicted gene targets.

To examine additional mechanisms via which LNA-antimiR-154 might protect the stressed heart we interrogated predicted gene targets of miR-154 using TargetScan Mouse 6.2, and performed pathway enrichment analysis (Partek® Genomics Suite v6.5). Consistent with miR-154 playing a key role in regulating fibrosis, the transforming growth factor beta (TGFβ) and Wnt signaling pathways were significantly enriched (Fig. 4, P = 0.02 and P = 0.04, respectively)^20^,^24^,^25^. In addition, the “Regulation of autophagy” pathway was enriched (Fig. 4, P < 0.02).

### miR-154 does not regulate autophagy in the heart

**Figure 5:**
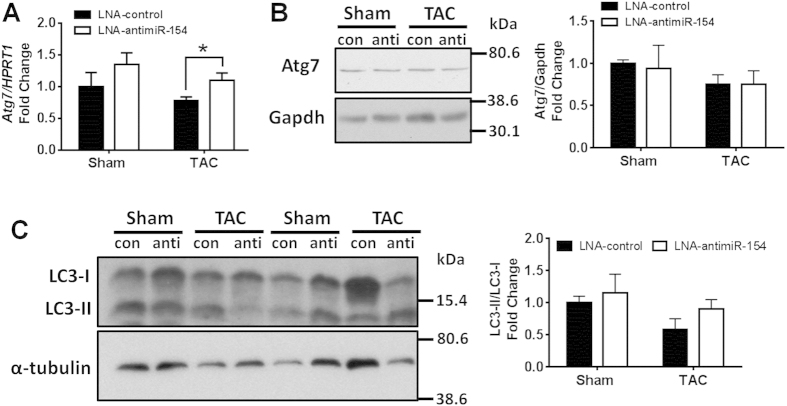
Atg7 is a predicted target of miRNA-154. (**A**) qPCR analysis of *Atg7* standardized to *Hprt1* in sham and TAC LNA-control and LNA-antimiR-154 treated mice. Data are expressed as mean ± SEM. N = 3–7/group. *P < 0.05. Unpaired t-test. (**B**) Representative Western blots and quantification of Atg7 in sham and TAC LNA-control (con) and LNA-antimiR-154 (anti) treated hearts. (**C**) Representative Western blots and quantification of LC3-II relative to LC3-I in sham and TAC LNA-control (con) and LNA-antimiR-154 (anti) treated hearts. Data are expressed as mean ± SEM.N = 3–5/group.

Since the “Regulation of autophagy” pathway was enriched and autophagy-related protein 7 (Atg7) is a predicted target of miR-154, we assessed the potential contribution of autophgy. Autophagy is an essential cellular process that degrades and recycles senescent or damaged proteins and organelles, and is required for normal homeostasis of cardiomyocytes^26^. In the present study, whilst mRNA expression of *Atg7* was increased in the hearts of LNA-antimiR-154 treated TAC mice compared with LNA-control-treated TAC mice (Fig. 5A), this was not accompanied by an increase in ATG7 protein expression (Fig. 5B). In addition, protein expression of another autophagic marker, LC3-II was not significantly different between TAC LNA-control and TAC LNA-antimiR-154 treated hearts (Fig. 5C). Thus, it is unlikely that miR-154 regulates autophagy and contributes to the cardiac phenotype, at least at the time point examined.

### LNA-antimiR-154 treatment was associated with less fibrosis

**Figure 6:**
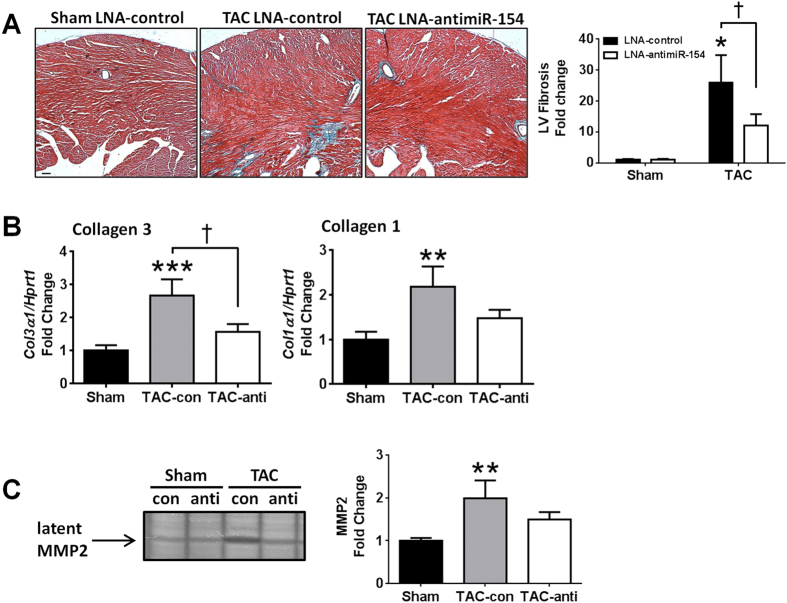
Inhibition of miR-154 was associated with less fibrosis and a favorable molecular gene signature. (**A**) Representative left ventricular cross-sections stained with Masson’s trichrome from sham LNA-control, TAC LNA-control, and TAC LNA-antimiR-154 mice, and quantification of fibrosis. Scale bar = 100 μM. Data are expressed as mean ± SEM. N = 5–8/group. *P  < 0.05 vs. sham of the same treatment group; ^†^P < 0.05. (**B**) qPCR analysis of *collagen 1 (Col1α1)* and *collagen 3 (Col3α1)* standardized to *Hprt1* in sham and TAC LNA-control and LNA-antimiR-154 treated mice. Data are expressed as mean ± SEM. N = 6–10/group. ***P < 0.001 vs. sham; **P < 0.01 vs. sham; *P < 0.05 vs. sham; ^†^P < 0.05. (**C**) Representative image of MMP2 by gelatin zymography in sham and TAC LNA-control (con) and LNA-antimiR-154 (anti) hearts. **P < 0.01 vs. Sham. N = 3–5/group. For panels (**B**,**C**) sham groups were combined because there were no differences between sham LNA-control and sham LNA-antimiR-154.

Previous studies in the lung^20^ and pathway enrichment analysis (Fig. 4) implicated miR-154 in regulating fibrosis. Fibrosis is a common feature of the failing myocardium and this is typically associated with increased levels of profibrotic factors and extracellular matrix proteins^27^. On histological analysis, hearts of TAC LNA-control mice displayed increased deposition of fibrosis (Fig. 6A) and this was accompanied by increased expression of collagen 3 (*Col3α1),* collagen 1 (*Col1α1*) (Fig. 6B), and latent MMP2 by gelatin zymography (Fig. 6C). These features were all attenuated by treatment with LNA-antimiR-154 (Fig. 6A–C).

**Figure 7:**
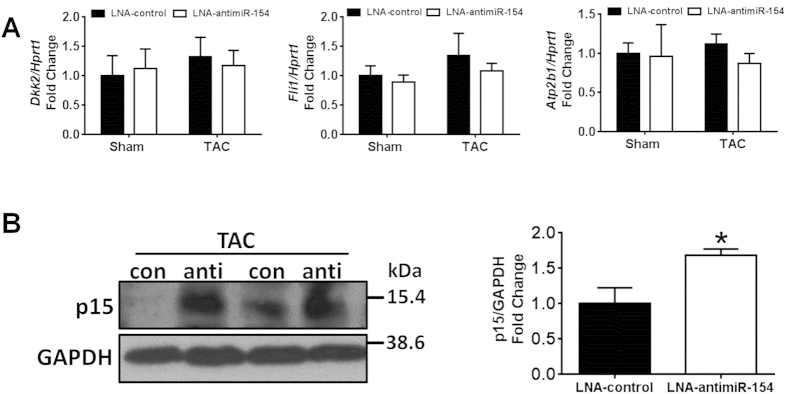
Analysis of miR-154 predicted targets. (**A**) qPCR analysis of miR-154 target genes including Dickkopf-related protein 2 *(Dkk2)*; Friend leukemia integration-1 *(Fli1)* and ATPase, Ca++ Transporting, Plasma Membrane 1 *(Atp2b1)* standardized to *Hprt1* in sham and TAC LNA-control and LNA-antimiR-154 treated mice. Data are expressed as mean ± SEM. N = 3–8/group. (**B**) Quantification and representative Western blot of p15 standardized to GAPDH in TAC LNA-control (con) and TAC LNA-antimiR-154 (anti) treated mice. Data are expressed as mean ± SEM. *P < 0.05 vs. LNA-control. N = 3/group.

We next investigated miR-154 targets which had been implicated in being involved with liver fibrosis including Dickkopf-related protein 2 *(Dkk2)*^28^, Friend leukemia integration-1 *(Fli1)* that has been implicated in extracellular matrix (ECM) gene regulation in skin fibroblasts^29^, a novel predicted target involved in calcium transporting, ATPase, Ca++ Transporting, Plasma Membrane 1 *(Atp2b1)*, and cyclin-dependent kinase inhibitor 2B *(Cdkn2b),* also known as p15, a cell cycle inhibitor shown to be decreased with lung fibrosis^20^. The gene expression of *Dkk2*, *Fli1* and *Atp2b1* did not change with LNA-antimiR-154 treatment (Fig. 7A). Protein expression of p15 increased with LNA-antimiR-154 treatment in TAC hearts compared to TAC LNA-control hearts (Fig. 7B). This suggests a potential mechanism by which miR-154 may regulate fibrosis in the heart.

### Chronic inhibition of miR-154 was not associated with morphological disarray

Consistent with the long half-life of LNA-antimiRs, inhibition of miR-154 was achieved in adult mouse organs including the heart, kidney, liver and lung, 8 weeks after LNA-oligonucleotide administration (Supplementary Fig. S3A). Chronic inhibition of miR-154 had no effect on liver organ weight in sham and TAC mice administered LNA-antimiR-154 compared to LNA-control mice (Supplementary Fig. S3B), and the heart and liver displayed no evidence of morphological disarray by histological assessment (Supplementary Fig. S3C). Due to the reported role of miR-154 in a setting of lung disease^20^, and the known crosstalk between the lungs and heart in disease settings^30^, we measured miR-154 in lungs of our disease model. Expression of miR-154 was not elevated in the lungs of TAC LNA-control mice compared with sham mice (Supplementary Fig. S4).

## Discussion

Numerous studies have highlighted the key role of miRNAs contributing to processes leading to HF, including cardiac hypertrophy, apoptosis and fibrosis^12^,^14^,^23^,^31^. Inhibition of pathogenic miRNAs with LNA-antimiRs has had a beneficial effect on cardiac function and pathology in animal models of HF^15^,^16^,^17^,^18^. We previously identified miRNAs that were differentially regulated in settings of cardiac stress and protection^4^, and demonstrated this as a valuable approach in identifying and targeting candidate miRNAs as a therapy for HF^15^,^16^,^17^. In the current study, we examined the effect of silencing miR-154 in a TAC mouse model which is characterized by hypertrophy, fibrosis and cardiac dysfunction. miR-154 was an appealing candidate as we previously reported that miR-154 expression was decreased in hearts of mice with increased PI3K activity, and increased in the diseased heart due to MI^4^. Furthermore, other investigators reported that the expression of miR-154 was elevated in two mouse models with established pathological hypertrophy due to TAC or cardiac-specific transgenic over-expression of activated calcineurin A^32^. Finally, it had previously been shown that miR-154 expression was increased in lungs of patients with pulmonary fibrosis, and a miR-154 inhibitor was able to attenuate TGFβ induced proliferation of human lung fibroblasts^20^. However, the role of miR-154 had not previously been examined in an *in vivo* model. This study represents the first to directly assess the role of miR-154 in a setting of pathological hypertrophy associated with fibrosis. Fibrosis is a common feature of heart disease of various aetiologies. Thus, the identification and development of therapies with antifibrotic properties is of great interest^23^,^33^,^34^.

Pressure overload is associated with numerous cardiac adaptive and maladaptive changes and responses including cardiac myocyte hypertrophy, fibrosis, autophagy and inflammation^35^. Over time, these features lead to pathological heart enlargement and cardiac dysfunction which progresses to heart failure. In the present study, treatment with LNA-antimiR-154 was associated with favourable outcomes in TAC mice including attenuation of heart size, lower atrial and lung weights, and preserved cardiac function. To explore potential mechanisms by which miR-154 may mediate protection in a setting of pathological hypertrophy, we investigated the effect of LNA-antimiR-154 on cardiomyocyte size, cardiac fibrosis, and autophagy. Here, we show that LNA-antimiR-154 mediated protection was associated with anti-hypertrophic actions and anti-fibrotic properties. Cardiac hypertrophy was examined by calculating LV mass during the time course of the study by echocardiography, as well as measuring heart size, cardiomyocyte size and hypertrophic genes (ANP and BNP) at study end. Collectively, the attenuation in cardiac enlargement, smaller myocyte size, and the absence of significant increases in ANP and BNP demonstrate that LNA-antimiR-154 treatment attenuated TAC-induced hypertrophy. Based on histological assessment of collagen deposition, MMP2 abundance and gene expression of extracellular markers, LNA-antimiR-154 treatment also attenuated TAC-induced fibrosis. To determine how silencing miR-154 could mediate antifibrotic effects in the heart, we investigated the expression of a number of miRNA gene targets previously linked with pulmonary or liver fibrosis^20^,^28^. Here we show that the protein expression of the cell cycle inhibitor, p15 (which is decreased in a setting of lung fibrosis) was increased with LNA-antimiR-154 treatment in TAC hearts compared to TAC LNA-control hearts. Thus, the regulation of p15 by miR-154 represents one mechanism by which LNA-antimiR-154 is likely to mediate anti-fibrotic effects in the heart.

We also investigated the possibility that miR-154 may mediate protection by regulating autophagy because Atg7, a predicted target of miR-154, is a key autophagy protein that had been shown to protect the stressed heart^36^. Hearts from transgenic mice with increased Atg7 expression had enhanced autophagy without heart pathology and cardiac dysfunction^36^. Furthermore, when Atg7 transgenic mice were bred with a mouse model with reduced autophagy and pathological remodelling, sustained Atg7-induced autophagy in the heart decreased fibrosis, attenuated pathological cardiac remodelling and improved survival^36^. In the current study, despite increased *Atg7* gene expression with LNA-antimiR-154 treatment, this was not accompanied by increased Atg7 protein expression. In addition, LC3 protein expression was not different between LNA-control and LNA-antimiR-154 treated hearts. Thus, it would appear autophagy has not contributed to the improved cardiac outcome in LNA-antimiR-154 TAC mice.

miRNAs are often associated in clusters or families, and miR-154 is highly conserved between species (Supplementary Fig. S5A). Several studies have shown a role for clustered miRNAs in disease^18^,^37^, and the therapeutic potential of targeting miRNA clusters and families in cancer and cardiovascular disease^16^,^18^,^38^. The therapeutic capacity of LNA-antimiR-154 may be increased by targeting other miRNAs that are mapped to the human chromosome 14q32 cluster, where miR-154 is located^20^,^39^. Expression of miR-495 and miR-299-5p, both localized to the chromosome 14q32 cluster, were also upregulated in a setting of MI and inversely correlated to cardiac function (Supplementary Fig. S5B,C)^4^. Thus, further improvements in cardiac function and pathology may be observed if multiple members of the 14q32 cluster are inhibited. This could be explored in future studies.

Finally, since i) miR-154 plays a role in lung disease^20^, ii) there was a moderate increase in lung weight in TAC LNA-control mice (Fig. 2C), and iii) organ crosstalk between the heart and lungs exists in disease settings^30^, it was considered possible that LNA-antimiR-154 therapy may have indirectly affected cardiac function via an effect in the lungs. To examine this possibility, we assessed miR-154 expression in the lungs of sham and TAC LNA-control mice. As miR-154 was not elevated in the lungs of our TAC model, we consider it unlikely that a decrease in miR-154 in the lungs of TAC LNA-antimiR-154 treated mice contributed to the cardiac phenotype. However, examining a potential lung-heart interaction would be of interest in more severe cardiac disease models in which miR-154 is elevated in the lungs.

**Figure 8:**
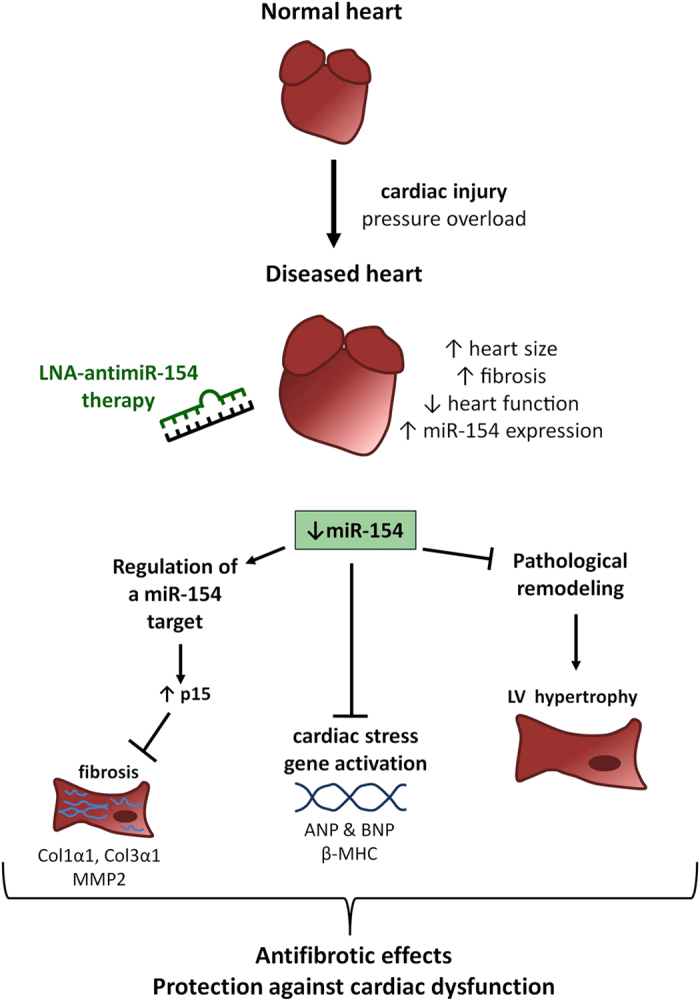
Potential protective mechanisms of antimiR-154 therapy. Schematic highlighting the major findings and potential mechanisms by which miR-154 inhibition may mediate protection in a setting of cardiac stress.

In conclusion, we show that *in vivo* subcutaneous delivery of LNA-antimiR-154 offers a promising therapeutic approach in a model of pathological hypertrophy associated with cardiac fibrosis. This study is the first to show that inhibition of miR-154 is able to attenuate cardiac hypertrophy, maintain cardiac function and protect against fibrosis. Mechanisms via which silencing miR-154 has antifibrotic properties and protects against cardiac dysfunction are highlighted in Fig. 8. Fibrosis is a pathological hallmark of chronic organ failure, including disorders of the heart, kidney, liver and lung, and chronic autoimmune diseases (e.g. scleroderma)^33^. Fibrotic disorders are associated with significant morbidity and mortality worldwide and there are limited effective therapies^22^. Since fibrosis is a common feature of numerous cardiac conditions which progress to HF including MI, hypertrophic cardiomyopathy and dilated cardiomyopathy, as well as being a predominant feature in a number of diseases in other organs^22^,^33^,^40^, inhibition of miR-154 using LNA therapeutics may aid in the treatment of numerous diseases associated with organ fibrosis.

## Materials and Methods

### Experimental animals

All experiments using animals were conducted in accordance with the Australian code for the care and use of animals for scientific purposes (National Health & Medical Research Council of Australia, 8^th^ Edition, 2013). Animal care and experimental procedures were approved by the Alfred Medical Research and Education Precinct’s Animal Ethics Committee.

### Pressure overload

Adult (~12 week old) male C57BL/6 mice were subjected to TAC (n = 15) or a sham (n = 6) operation as previously described^8^. The TAC model induces a chronic pressure load on the heart and is associated with progressive pathological hypertrophy and cardiac dysfunction within four weeks of surgery^8^. Briefly, prior to surgery, mice were anaesthetized with a combination of ketamine, xylazine and atropine (100:20:1.2 mg/kg, i.p.), administered an analgesic (carprofen, 5 mg/kg, s.c.) and intubated for ventilation. Mice were then administered a local anaesthetic (lignocaine, 7 mg/kg, s.c.) at the site of incision. A sternectomy was performed to access the aorta and a non-absorbable 5-0 braided silk suture was tied around the aorta between the right innominate and left carotid arteries, causing a constriction of approximately 65% using a 0.46 mm probe as a guide. For sham operations to serve as controls, mice (sham, n = 6) received the same surgical procedure except that the suture around the aorta was removed prior to closing the chest cavity.

### LV structure and function

Transthoracic echocardiography (2D and M-mode echocardiography) was performed on anaesthetized mice (1.8% isoflurane, inhalation) using a Philips iE33 ultrasound machine with a 15 mHz liner array transducer, prior to surgery (baseline), 4 weeks post-TAC, and 8 weeks post-treatment (i.e. 12 weeks post-TAC, endpoint). LV chamber dimensions (LV end-diastolic dimension, LVEDD; LV end-systolic dimension, LVESD), LV wall thicknesses (LV posterior wall, LVPW; interventricular septum, IVS), heart rate (HR), LV mass [calculated as 1.05 × ([[LVEDD + LVPW + IVS]^3^−[LVEDD]^3^/1000])^41^ and fractional shortening (FS, calculated as [(LVEDD – LVESD)/LVEDD] × 100%) were analysed offline using dedicated software (ProSolv Cardiovascular Analyzer version 3.5; ProSolv, Indianapolis, IN, USA). Each parameter was obtained from measuring 3 beats from M-mode images for each mouse in each group.

### LNA oligonucleotides

The miRCURY LNA™ microRNA Inhibitor (mmu-miR-154-5p and a scrambled control) is LNA™-enhanced (LNA/DNA mixmer with ~50% LNA content) and contains a phosphorothioate backbone (Exiqon, Skelstedet, Denmark). LNA-oligonucleotides were purified and analysed using anion-exchange high-performance liquid chromatography, desalted and lyophilized as a sodium salt. LNA-oligonucleotides were resuspended in saline at 5 mg/ml, aliquoted and stored at −20 °C. The sequence for mmu-miR-154-5p antimiR is 5′CAACACGGATAACCT 3′ (Batch Number 508003) and for the scrambled control 5′ACGTCTATACGCCCA 3′ (Batch Number 508005).

### *In vivo* delivery of LNA-antimiR oligonucleotides

Following 4 weeks of pressure overload, all mice underwent echocardiography to confirm the degree of LV remodelling and cardiac dysfunction. Sham and TAC mice were then randomized into control or treated groups. The TAC groups had a similar degree of LV hypertrophy based on LV wall thickness and LV mass after 4 weeks of pressure overload, prior to commencement of treatment. Mice were subcutaneously administered LNA-control or LNA-antimiR-154 (25 mg/kg/day) over three consecutive days and left for a period of 8 weeks (Supplementary Fig. S2). Our previous studies demonstrated that 3 consecutive daily subcutaneous injections of an LNA-antimiR was sufficient to inhibit microRNA gene expression for at least 2 months^15^,^16^,^17^.

An additional subset of unoperated, aged matched male mice (n = 13) were included within the study. Since the sham operation had no effect on morphological, functional or biochemical parameters, unoperated and sham mice were combined and randomized into saline/LNA-control or LNA-antimiR-154 treated groups. For clarification, the “sham LNA-control group” includes 3 sham LNA-control mice and 4 unoperated saline mice, and the “sham LNA-antimiR-154 group” includes 3 sham LNA-antimiR-154 treated mice and 9 unoperated LNA-antimiR-154 treated mice.

### RNA isolation

Total RNA was isolated from frozen mouse tissues using TRI Reagent (Sigma-Aldrich, St Louis, MO, USA) and quantitated on a Nanodrop™ Spectrometer (Thermo Scientific, Waltham, MA, USA).

### Protein isolation

For protein lysates, frozen mouse ventricles were homogenized in a lysis buffer (10% glycerol; 137 mM NaCl; 20 mM Tris-HCl, pH 7.4; 20 mM NaF; 10 mM EDTA; 1 mM EGTA; 1 mM sodium pyrophosphate; 1 mM vanadate; 1 mM PMSF; 4 g/ml pepstatin; 4 g/ml aprotinin; and 4 g/ml leupeptin). Total protein concentration was determined using the Bradford assay (Bio-Rad, Hercules, CA, USA).

### Quantitative RT-PCR (qPCR)

For mRNA expression analysis, 2 μg of total RNA was reverse transcribed using the High Capacity RNA-to-cDNA kit (Life Technologies, Carlsbad, CA, USA) according to manufacturer’s recommendations. qPCR was performed using TaqMan® probes (Life Technologies) and amplified on an Applied Biosystems 7500 real-time PCR instrument according to manufacturer’s instructions. Hypoxanthine phosphoribosyltransferase 1 (*Hprt1)* was used to standardize for cDNA concentration and data was analysed using the 2^−∆∆Ct^ method of quantification. For miRNA expression analysis, 50–100 ng of total RNA was reversed transcribed using TaqMan® MicroRNA Reverse Transcription Kit (Life Technologies) according to manufacturer’s recommendations. qPCR was performed using TaqMan® MicroRNA Assays (Life Technologies), expression was normalized against snoU6 and data analysed using the 2^−∆∆Ct^ algorithm.

### SDS-Polyacrylamide gel electrophoresis (PAGE) and Western blotting

Heart protein lysate (20–100 μg) was separated by SDS-PAGE, transferred to PVDF membrane and probed overnight with primary antibodies for α-tubulin, (Cell Signaling Technology, Danvers, MA, USA, 2144 S,1:1000), Atg7 (Cell Signaling Technology, 8558,1:1000), GAPDH (Santa Cruz, Santa Cruz, CA, sc-32233, 1:5000), LC3 (Cell Signaling, 2775,1:1000) and p15 (Santa Cruz, sc-613, 1:500). Signals were detected by chemiluminescence and quantified using ImageJ 1.44p pixel analysis (US National Institutes of Health).

### Gelatin zymography

To asses MMP2 abundance, 100 μg of heart protein lysate was separated by SDS gelatin gel. The gel was washed in 0.25% Triton X-100 for 30 min to remove excess SDS and then incubated overnight at 37 °C in incubation buffer (50 mM Tris (pH 7.4), 10 mM CaCl_2_, 1% Triton X-100, 0.02% NaN_3_, 1 μM ZnCl_2_). The gel was stained in 0.1% Coomassie blue for at least 1 h and then destained (7% acetic acid, 20% methanol) for 2–4 h. MMP2 bands were quantified using ImageJ 1.44p pixel analysis (US National Institutes of Health).

### Histological analyses

Tissue samples were fixed in 4% paraformaldehyde overnight and paraffin embedded for histological analysis at 6 μm cross-sections. To assess tissue morphology, sections were stained with haematoxylin and eosin (H&E). Cardiac collagen deposition/interstitial fibrosis (Masson’s trichrome stain) and cell area (wheat germ agglutinin stain, WGA) of ventricles were assessed as previously described^17^.

### Statistical Analyses

Statistical analyses were performed using StatView (Version 5.0.1, SAS Institute Inc., Cary, NC, USA). Results are presented as means ± SEM. For normally distributed data, differences between groups were identified using one-way analysis of variance (ANOVA) followed by *Fisher’s* post-hoc test. Unpaired t-tests were used when comparing two groups for a single measure. If data were not normally distributed, differences between groups were identified using a Nonparametric test (Mann-Whitney test). For echocardiography parameters, differences between groups were identified using a two-way repeated measures ANOVA followed by *Fisher’s* post-hoc test. A value of P < 0.05 was considered significant. All relative units are expressed as a fold change with the relevant control group normalised to 1.

## Additional Information

**How to cite this article**: Bernardo, B. C. *et al.* Inhibition of miR-154 Protects Against Cardiac Dysfunction and Fibrosis in a Mouse Model of Pressure Overload. *Sci. Rep.* **6**, 22442; doi: 10.1038/srep22442 (2016).

## Supplementary Material

Supplementary Information
