# Supplementary material for: Inhibition of miR-154 Protects Against Cardiac Dysfunction and Fibrosis in a Mouse Model of Pressure Overload
Source: Sci Rep. 2016 Mar 1;6:22442. doi: 10.1038/srep22442 (PMC4772383; doi:10.1038/srep22442)
Supplement: Supplementary Information [file srep22442-s1.pdf]

SUPPLEMENTARY INFORMATION

Inhibition of miR-154 Protects Against Cardiac Dysfunction and Fibrosis in a Mouse Model of Pressure Overload

Bianca C. Bernardo[1*], Sally S. Nguyen[1,2], Xiao-Ming Gao[1], Yow Keat Tham[1,3], Jenny Y.Y. Ooi[1], Natalie L. Patterson[1], Helen Kiriazis[1], Yidan Su[1], Colleen J. Thomas[2], Ruby C. Y. Lin[4,5], Xiao-Jun Du[1,3] and Julie R. McMullen[1,3*]

[1]Baker IDI Heart and Diabetes Institute, Melbourne, 3004, Australia
[2]Department of Physiology, Anatomy and Microbiology, La Trobe University, Bundoora, 3086, Australia
[3]Monash University, Clayton, 3800, Australia
[4]Asbestos Diseases Research Institute, Concorde Hospital, 2139, Australia
[5]Ramaciotti Centre for Genomics and School of Biotechnology and Biomolecular Sciences, University of New South Wales, Sydney, 2052, Australia

To whom correspondence should be addressed: *Bianca C. Bernardo: PO Box 6492, Melbourne 3004, Australia. Tel: +61 3 8532 1167, Fax: +61 3 8532 1100, E-mail: bianca.bernardo@bakeridi.edu.au and Julie R. McMullen: PO Box 6492, Melbourne 3004, Australia. Tel: +61 3 8532 1194, Fax: +61 3 8532 1100, E-mail: julie.mcmullen@bakeridi.edu.au

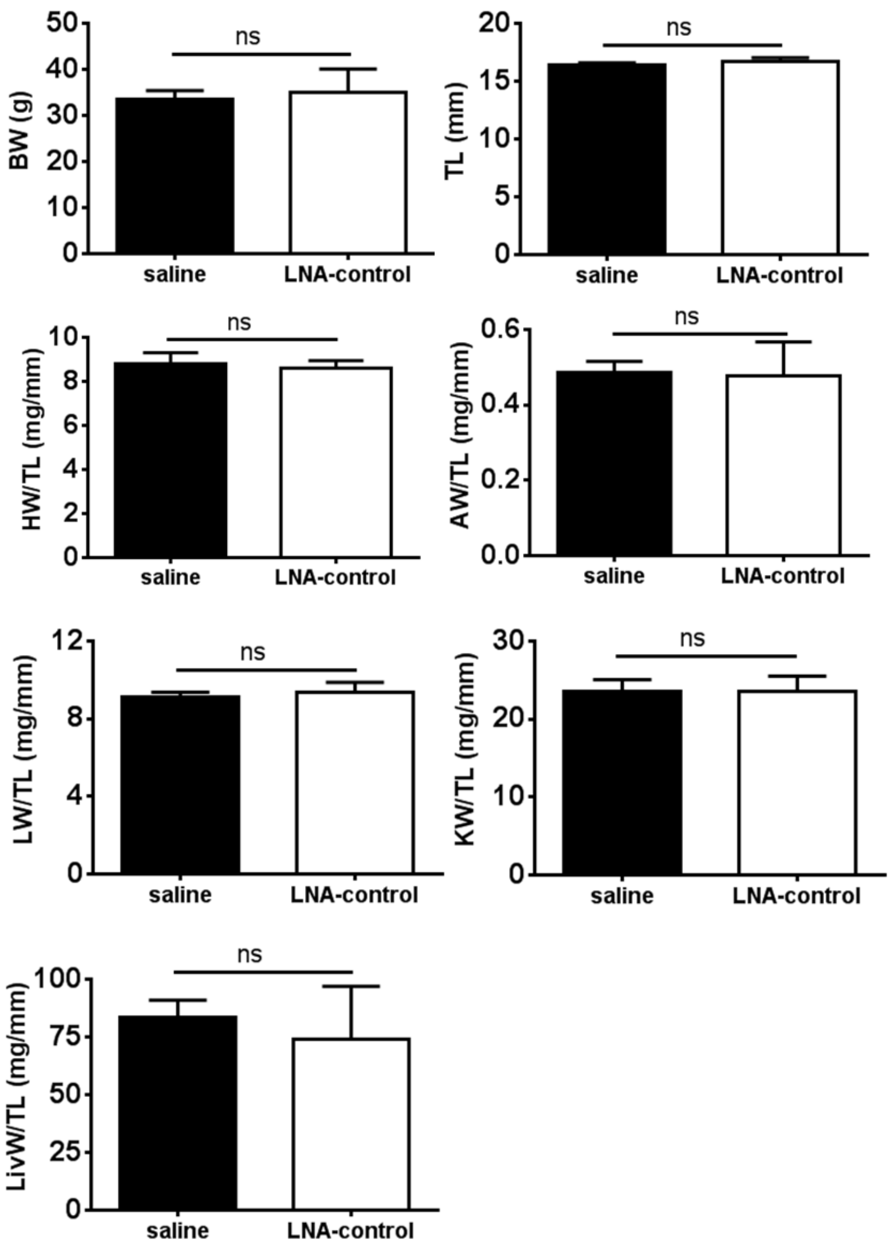

Supplementary Figure S1. Analysis of body weight, tibial length and organ weights in adult sham or unoperated, aged matched male C57BL/6 mice treated with saline or LNA-control. Unpaired t-test. N=3-4/group. ns = not significant. BW, body weight; TL, tibia length; HW, heart weight; AW, atrial weight; LW, lung weight; LivW, liver weight; KW, kidney weight.

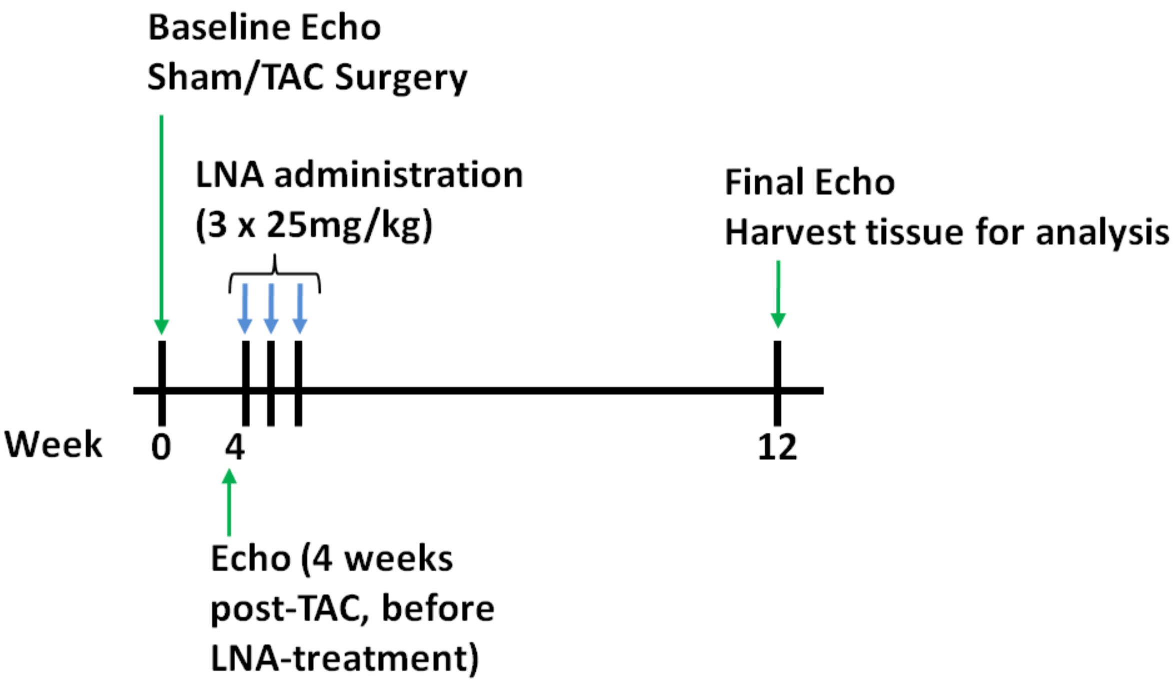

Supplementary Figure S2. Experimental Timeline and dosing regimen of LNA-control/antimiR-154 for mice subjected to sham or pressure overload (TAC). Echo = echocardiography, LNA = locked nucleic acid, TAC = transverse aortic constriction

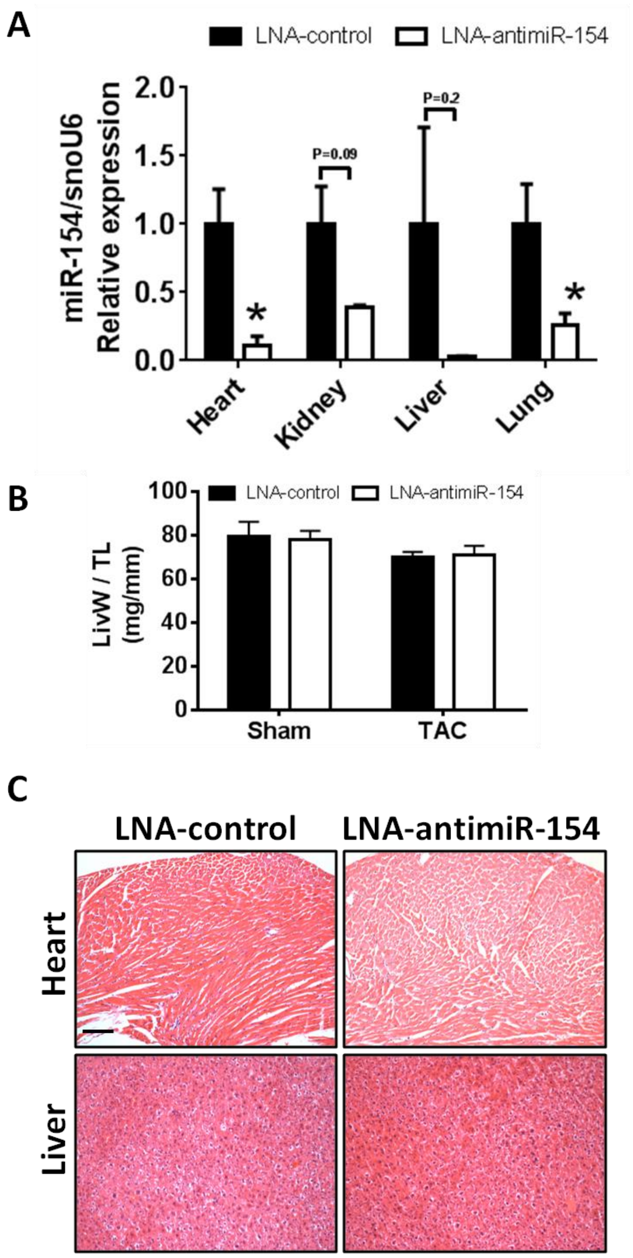

Supplementary Figure S3. Chronic inhibition of LNA-antimiR-154 was not associated with adverse effects in other tissues based on morphology and histology. A) Inhibition of miR-154 relative to snoU6 by qPCR in heart, kidney, liver, lung, skeletal muscle and spleen from sham adult mice treated with LNA-control or LNA-antimiR-154 as assessed by qPCR. Data are expressed as mean±SEM. N=3-4/group. *P<0.05 vs. LNA-control. Unpaired t-test. **B)** Kidney weight/TL (KW/TL), Liver weight/TL (LivW/TL) and Spleen weight/TL (SW/TL) in sham and TAC mice treated with LNA-control or LNA-antimiR-154. Data are expressed as mean±SEM. N=3-8/group. **C)** Representative heart (LV), kidney, liver and lung sections from LNA-control and LNA-antimiR-154 treated mice (8 weeks after LNA-antimiR delivery) stained with H&E. Scale bar=100 μm.

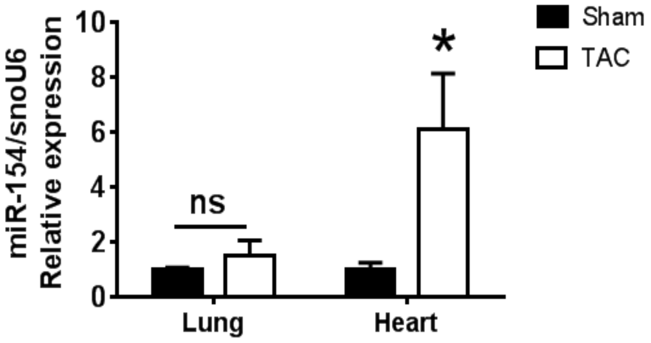

Supplementary Figure S4. miR-154 expression in lung. Expression of miR-154 relative to snoU6 by qPCR in lung from sham and TAC adult mice treated with LNA-control. Data are expressed as mean±SEM. N=5-6/group. *P<0.05 vs. sham. Unpaired t-test. ns=not significant. Heart (from Fig 2A in main text) is included as a comparison to demonstrate significant elevation of miR-154 in the heart in response to TAC.

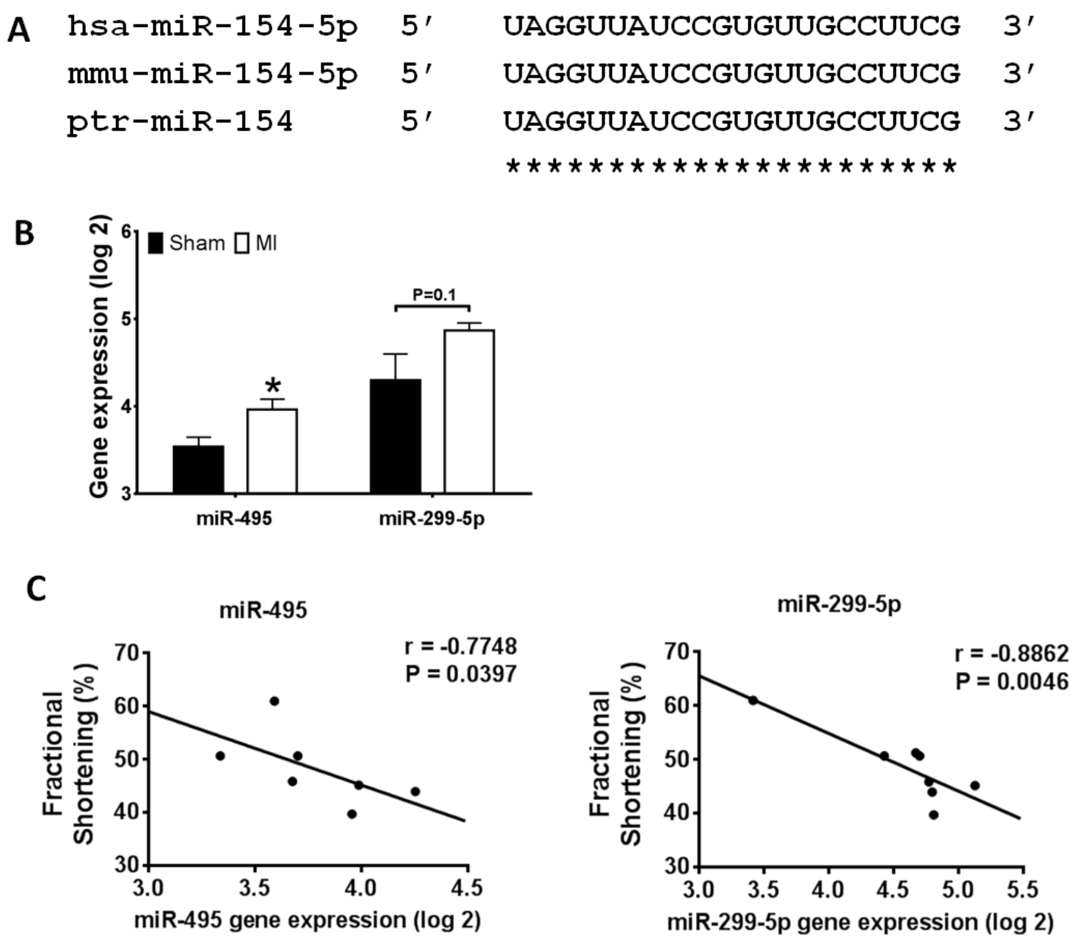

Supplementary Figure S5. Analysis of miR-495 and miR-299-5p in sham and MI mice. **A)** Sequence alignment of human (hsa), mouse (mmu) and chimpanzee (ptr) showing complete conservation of miR-154 between species (same base pairs are indicated by *) **B)** Gene expression (log 2, normalized sequencing reads) of miR-495 and miR-299-5p by microarray in hearts from Ntg sham and MI mice (1). Data are expressed as mean±SEM. N=3-4 per group. Unpaired t-test. **C)** Linear correlation between miR-495 and miR-299-5p gene expression (log 2) in hearts from mice subjected to sham or MI from microarray analysis and cardiac function (fractional shortening) (1). N=7-8/group. P<0.05 (Spearman's correlation).

Supplementary Table S1: Morphological data of sham and TAC LNA-control and LNA-antimiR-154 treated mice following 8 weeks of treatment.

	Sham		TAC	
	**LNA-control**	**LNA-antimiR-154**	**LNA-control**	**LNA-antimiR-154**
**No. of animals**	7	12	7	8
**Body weight (g)**	34.1±1.6	33.1±0.5	31.2±1.4	32.3±1.0
**Tibial length (mm)**	16.5±0.1	16.3±0.1	16.4±0.1	16.6±0.1
**Heart weight (mg)**	144.4±5.1	142.3±5.3	210.2±19.4***	185.5±14.5**
**Atrial weight (mg)**	8.0±0.4	8.3±0.6	14.4±2.6**	11.2±1.5
**Lung weight (mg)**	152.6±3.3	145.1±4.1	234.8±47.6*	166.2±4.6†
**HW/BW (mg/g)**	4.3±0.2	4.3±0.1	6.8±0.7***	5.7±0.4**‡
**AW/BW (mg/g)**	0.24±0.02	0.25±0.02	0.47±0.1**	0.35±0.05‡
**LW/BW (mg/g)**	4.5±0.2	4.4±0.1	7.8±1.7**	5.2±0.2†
**HW/TL (mg/mm)**	8.7±0.3	8.7±0.3	12.8±1.1***	11.2±0.9**‡
**AW/TL (mg/mm)**	0.48±0.03	0.51±0.04	0.87±0.13**	0.67±0.09‡
**LW/TL (mg/mm)**	9.2±0.2	8.9±0.2	14.3±2.9*	10.0±0.3†
**Liver weight (mg)**	1315.1±116.2	1267.9±63.6	1148.3±41.8	1177.7±67.1
**LivW/BW (mg/g)**	38.3±2.0	38.5±2.0	37.3±2.4	36.7±2.4
**LivW/TL (mg/mm)**	79.4±6.7	77.7±4.1	69.8±2.4	70.8±4.1

BW, body weight; TL, tibial length; HW, heart weight; AW, atrial weight; LW, lung weight; LivW, liver weight. Data are shown as mean ± SEM. One-way ANOVA followed by *Fisher's* post-hoc test. *P<0.05 vs. sham of the same treatment group,**P<0.01 vs. sham of the same treatment group, ***P<0.001 vs. sham of the same treatment group, †P<0.05 vs. TAC LNA-control, ‡P≤0.1 vs. TAC LNA-control (HW/BW P=0.06; HW/TL P=0.1, AW/TL=0.1, AW/BW=0.09).

Supplementary Table S2: Echocardiography measurements of sham and TAC LNA-control and LNA-antimiR-154 treated mice at baseline, 4 weeks post-TAC and 8 weeks post-treatment.

	Baseline				4 weeks post-TAC				12 weeks post-TAC			
	Sham		TAC		Sham		TAC		Sham		TAC	
	control	antimiR-154	control	antimiR-154	control	antimiR-154	control	antimiR-154	control	antimiR-154	control	antimiR-154
**No. of animals**	3	3	7	8	3	3	7	8	3	3	7	8
**BW, g**	29.0±1.9	26.4±0.7	26.8±0.6	27.7±0.6	30.0±2.3	28.5±0.6¶	29.2±0.8¶	29.7±0.7¶	35.7±2.8¶	32. 9±1.6¶	31. 5±1.3¶	32.8±1.0¶
**HR, bpm**	484±26	537±29	545±17	527±20	598±21	560±53	551±14	591±20‖	579±13	590±12	565±9	583±15‖
**LVPW, mm**	0.73±0.03	0.75±0.01	0.73±0.01	0.75±0.01	0.79±0.01	0.79±0.01	1.05±0.01*	1.04±0.02*	0.83±0.01	0.80±0.01	1.10±0.02*	1.13±0.02*‡
**IVS, mm**	0.79±0.03	0.80±0.01	0.76±0.02	0.80±0.01	0.83±0.01	0.82±0.01	1.14±0.02*	1.12±0.01*	0.85±0.01	0.85±0.01	1.15±0.02*	1.18±0.02*‡
**LVEDD, mm**	4.07±0.20	3.83±0.15	4.11±0.13	3.89±0.13	3.81±0.06	3.74±0.06	4.09±0.10	3.84±0.12	3.46±0.17	3.46±0.14	4.33±0.29	3.59±0.08†
**LVESD, mm**	2.33±0.22	2.15±0.20	2.34±0.14	2.29±0.10	1.97±0.09	2.21±0.13	2.83±0.08*	2.57±0.10	1.83±0.05	1.90±0.08	3.22±0.34*§	2.31±0.12†
**LV mass, g**	0.113±0.004	0.104±0.007	0.113±0.005	0.108±0.006	0.110±0.003	0.106±0.002	0.189±0.008*	0.169±0.009*	0.099±0.009	0.096±0.006	0.216±0.020*‡	0.167±0.007*†
**FS, %**	43±2	44±3	43±2	41±1	48±2	41±3	31±1*	33±1*	47±3	45±3	27±3*§	36±2*†

BW, body weight; HR, heart rate; LV, left ventricular; LVPW, LV posterior wall thickness; IVS, interventricular septum thickness; LVEDD, LV end-diastolic dimension; LVESD, LV end-systolic dimension; FS, fractional shortening. Data are shown as mean ± SEM. Two Way Repeated Measures ANOVA with *Fisher's* post-hoc test. *P<0.05 vs. sham of the same treatment at the same timepoint, and baseline of the same group; †P<0.05 vs. TAC LNA-control at same timepoint; ‡P<0.05 vs. same group at 4 weeks post-TAC; §P=0.07 vs. same group at 4 weeks post-TAC; ‖P<0.05 vs. baseline of same group only; ¶ P<0.05 vs. baseline of the same group, and 12 weeks post-TAC compared to 4 weeks post-TAC of the same group.

References

1. Lin, R. C. Y., Weeks, K. L., Gao, X.-M., Williams, R. B. H., Bernardo, B. C., Kiriazis, H., Matthews, V. B., Woodcock, E. A., Bouwman, R., Mollica, J. P., Speirs, H. J., Dawes, I. W., Daly, R. J., Shioi, T., Izumo, S., Febbraio, M. A., Du, X.-J., and McMullen, J. R. (2010) PI3K(p110α) protects against myocardial infarction-induced heart failure/ Identification of PI3K-regulated miRNAs and mRNAs. *Arterioscler. Thromb. Vasc. Biol.* **30**, 724-732
